# Integrative management of gastric cardia cancer and precancerous lesions: prospects and mechanistic rationale for Banxia Xiexin Tang

**DOI:** 10.3389/fphar.2026.1763396

**Published:** 2026-06-19

**Authors:** Xiao Liang, Liu Yang, Yueshan Zhang, Shuaiqi Yuan, Yonggui Hong

**Affiliations:** Anyang Tumor Hospital, The Affiliated Anyang Tumor Hospital of Henan University of Science and Technology, Anyang, Henan, China

**Keywords:** Banxia Xiexin Tang, gastric cardia cancer, gastroesophageal junction, integrative oncology, standard biomedical treatment, Traditional Chinese Medicine

## Abstract

Gastric cardia cancer is a biologically heterogeneous malignancy arising at the gastroesophageal junction that is, the upper opening of the stomach rather than the heart. It develops along an inflammation-metaplasia-dysplasia-carcinoma sequence and remains clinically challenging because early lesions are often occult and advanced disease is shaped by a treatment-resistant tumor microenvironment. A clinically useful review therefore needs to distinguish gastric cardia cancer from non-cardia gastric cancer and from esophageal adenocarcinoma, while also clarifying where current evidence is direct and where it is only extrapolated. Banxia Xiexin Tang (BXT) is a seven-botanical formula containing Pinellia ternata (Thunb.) Makino. [Araceae] (Pinelliae Rhizoma), Coptis chinensis Franch. [Ranunculaceae] (Coptidis Rhizoma), Scutellaria baicalensis Georgi [Lamiaceae] (Scutellariae Radix), Zingiber officinale Roscoe [Zingiberaceae] (Zingiberis Rhizoma Recens), Panax ginseng C.A.Mey. [Araliaceae] (Ginseng Radix et Rhizoma), Glycyrrhiza uralensis Fisch. ex DC. [Fabaceae] (Glycyrrhizae Radix et Rhizoma), and Ziziphus jujuba Mill. [Rhamnaceae] (Jujubae Fructus). Available preclinical and supportive-care studies suggest that Banxia Xiexin Tang or selected metabolites within the formula may modulate inflammatory signaling, oxidative stress, epithelial barrier injury, and treatment-related gastrointestinal toxicity. However, the current evidence base is uneven: much of it comes from gastritis, reflux injury, or non-cardia gastric disease models rather than directly from human gastric cardia cancer. This review critically synthesizes the available literature on integrative management of gastric cardia cancer and related precancerous lesions, with emphasis on what the current data do support, what they do not support, and what research gaps remain. The revision adds a transparent search strategy, clearer terminology, a more cautious interpretation of pharmacological claims, and an explicit discussion of limitations. Because the included studies were heterogeneous in design, population, intervention, and outcome reporting, the evidence is synthesized narratively rather than through quantitative meta-analysis.

## Graphical Abstract

**Figure:**
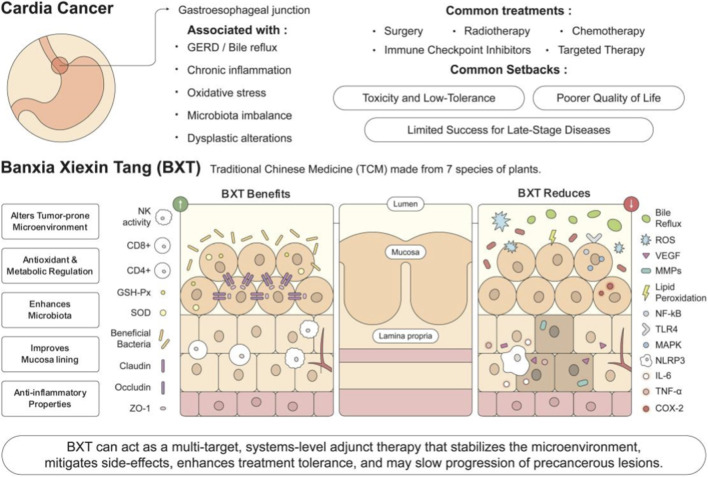

## 1 Introduction

Gastric cardia cancer is a distinct subtype of gastric carcinoma that develops at the anatomic transition between the distal esophagus and the proximal stomach, namely the gastroesophageal junction (GEJ) (Rüdiger Siewert et al., 2000; Lv et al., 2021). This region is exposed to refluxed acid, bile, microbial shifts, and chronic inflammation, which together create a biologically complex setting for carcinogenesis (He et al., 2022). Importantly, gastric cardia cancer should not be described as “cardiac cancer,” because the latter term is easily misunderstood as a malignancy of the heart.

From an anatomical and biological perspective, gastric cardia cancer shares features with both proximal gastric adenocarcinoma and adenocarcinoma arising near the GEJ, yet it is not identical to either disease category. In contrast to many non-cardia gastric cancers, cardia tumors are more strongly linked to reflux-related mucosal injury and junctional microenvironmental disruption. Their development typically involves chronic inflammation, intestinal metaplasia, dysplasia, and malignant transformation, with contributions from smoking, high-salt diets, nitrosamine exposure, and persistent oxidative stress.

**FIGURE 1:**
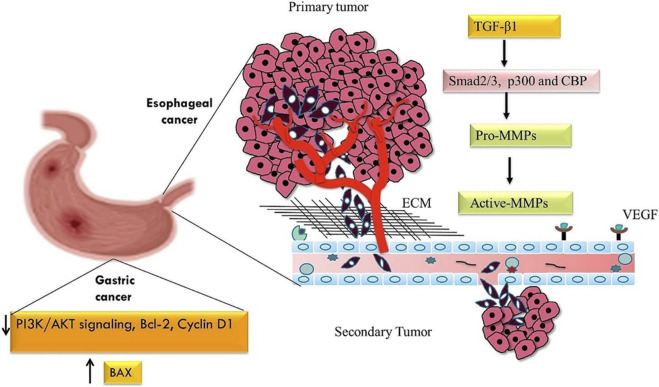
Illustration of the Cancer Pathway in the Stomach and Esophagus: This detailed diagram highlights the molecular mechanisms and key factors involved in the development and progression of cancer within the gastrointestinal tract. The pathway underscores critical mutations, signaling pathways, and cellular changes that contribute to the onset and metastasis of stomach and esophageal cancers (Patwa et al., 2024).

As such, the molecular pathways of oncogenic development (e.g., NF-κB, Wnt/β-catenin, EGFR, PI3K/AKT) are easily activated by this microenvironment. In addition to promoting tumor cell proliferation and anti-apoptotic pathways, the activation of these pathways promotes other important processes, including angiogenesis, epithelial-mesenchymal transition, and immune evasion. The relevant signal pathways are shown in Figure 1.

Cardia cancer therapy has been standardized with significant advancements over time and includes endoscopic mucosal resection for early stage lesions, perioperative chemotherapy, postoperative radiation and chemotherapy, targeted therapies (HER2, VEGF, Claudin-18.2), and most recently immune checkpoint inhibitors (Fattahi et al., 2020). Although many advances have been made in recent years, the prognosis for most patients with malignant diseases remains quite dismal, especially those diagnosed at a late stage of disease whose chances of survival are less than 25%. Both cytotoxic and immunotherapy regimens used in the treatment of malignancy have additive toxicities that limit patient compliance with prescribed therapy and decrease the overall quality of life experienced by the majority of patients with cancer. These factors have led to increasing interest in using alternative therapies as adjuvants to standard medical treatments to decrease toxicity from cancer therapy and potentially enhance the effectiveness of standard therapies.

Traditional Chinese Medicine (TCM) offers a whole-system framework for the management of cancer and precancerous states (Xu et al., 2022). In this framework, disease is interpreted as a dynamic imbalance involving digestive function, inflammatory burden, host resilience, and mucosal repair (Zhong et al., 2023). Such a perspective does not replace standard biomedical treatment; rather, it may complement surgery, endoscopy, systemic therapy, and supportive care when supported by reproducible evidence and appropriate safety oversight (Mao et al., 2022).

The integration of TCM and standard biomedical treatment is therefore most defensible when it is framed as evidence-informed supportive or adjunctive care rather than as an alternative to oncologic therapy (Greenlee et al., 2017). For gastric cardia cancer, the relevant scientific question is not whether a traditional formula is culturally familiar, but whether it has biologically plausible mechanisms, measurable clinical benefits, acceptable safety, and sufficient quality control to justify integration into contemporary oncology.

Banxia Xiexin Tang (BXT) is relevant to this discussion because it has long been used for upper gastrointestinal symptom patterns and because modern studies have linked the formula, or its constituent metabolites, to pathways involved in inflammation, epithelial injury, oxidative stress, and mucosal repair (Zhou et al., 2023). Banxia Xiexin Tang (BXT) is a seven-botanical formula containing Pinellia ternata (Thunb.) Makino. [Araceae] (Pinelliae Rhizoma), Coptis chinensis Franch. [Ranunculaceae] (Coptidis Rhizoma), Scutellaria baicalensis Georgi [Lamiaceae] (Scutellariae Radix), Zingiber officinale Roscoe [Zingiberaceae] (Zingiberis Rhizoma Recens), Panax ginseng C.A.Mey. [Araliaceae] (Ginseng Radix et Rhizoma), Glycyrrhiza uralensis Fisch. ex DC. [Fabaceae] (Glycyrrhizae Radix et Rhizoma), and Ziziphus jujuba Mill. [Rhamnaceae] (Jujubae Fructus) - whose major metabolites have been investigated in network pharmacology, experimental pharmacology, and translational gastrointestinal research (Li Y. et al., 2024).

Current preclinical evidence suggests that BXT may reduce pro-inflammatory mediators, improve antioxidant defenses, support tight-junction integrity, and modify upper gastrointestinal microbial ecology (Wang et al., 2021). These observations provide a mechanistic rationale for further study, but they should not be overstated: direct evidence in human gastric cardia cancer remains limited, and mechanistic findings from gastritis or reflux models cannot automatically be interpreted as proof of anti-cancer efficacy (Cao et al., 2020).

An integrative approach remains conceptually attractive because gastric cardia carcinogenesis is driven not only by malignant epithelial cells but also by inflammation, barrier disruption, reflux injury, stromal remodeling, immune dysfunction, and microbial imbalance. A multi-component formula such as BXT may therefore be relevant to the host-tumor microenvironment interface; however, this possibility must be judged through critical pharmacological and clinical assessment, not by narrative accumulation of favorable claims.

This review critically evaluates scientific, clinical, and translational evidence relevant to the integration of BXT with standard biomedical treatment for gastric cardia cancer and related precancerous lesions.

### 1.1 Review methodology and literature search

A structured literature review was conducted across PubMed, Embase, Web of Science, Cochrane Library, CNKI, Wanfang, and ClinicalTrials.gov. Searches targeted studies published from January 2020 to October 2025 and combined terms related to gastric cardia cancer, gastroesophageal junction, precancerous lesions, Banxia Xiexin Tang, traditional Chinese medicine, and integrative therapy by using Boolean operators AND/OR. English-language and Chinese-language publications were considered.

Eligible records included original preclinical studies, clinical studies, translational studies, and high-quality reviews that were directly relevant to gastric cardia cancer, upper gastric/GEJ precancerous lesions, BXT, or closely related integrative gastrointestinal oncology questions. Editorials, conference abstracts without usable data, duplicate reports, and studies without sufficient methodological detail were excluded. Titles and abstracts were screened first, followed by full-text assessment of potentially eligible studies. Data extraction focused on study design, population or model, intervention characteristics, comparator, outcome measures, and major limitations.

## 2 Pharmacological basis and mechanistic insights of Traditional Chinese Medicine

Traditional Chinese Medicine formulas are pharmacologically complex because multiple botanical drugs and their metabolites may interact with overlapping inflammatory, oxidative, epithelial, and immune pathways (Yang Y. et al., 2023). For BXT, the mechanistic rationale is biologically plausible, but the available literature is stronger for pathway directionality than for standardized quantitative pharmacology. The key question is therefore not whether each metabolite has any activity, but whether the complete formula has reproducible, well-characterized activity in models relevant to gastric cardia carcinogenesis and treatment support.

BXT comprises seven botanical drugs. Representative metabolites include berberine and coptisine from Coptidis Rhizoma, baicalin and baicalein from Scutellariae Radix, gingerols and shogaols from Zingiberis Rhizoma Recens, and ginsenosides from Ginseng Radix et Rhizoma (He et al., 2024). These metabolites have been associated in the literature with anti-inflammatory, antioxidant, epithelial-protective, and immunomodulatory effects. Nevertheless, activity reported for an isolated metabolite should not automatically be attributed to the whole BXT formula unless the complete decoction or a standardized BXT preparation was directly tested.

**FIGURE 2:**
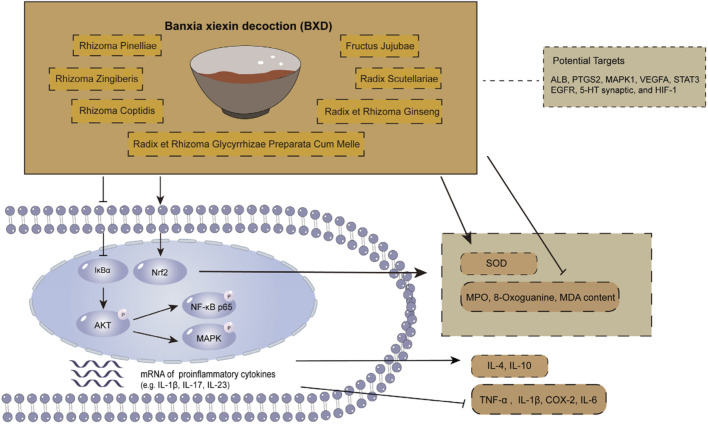
Mechanistic framework relevant to Banxia Xiexin Tang (BXT) in gastrointestinal inflammation and mucosal injury.

The mechanistic literature suggests several potentially relevant actions of BXT, including attenuation of TLR4/NF-kB-related inflammatory signaling, reduction of oxidative stress, reinforcement of epithelial barrier proteins, and modulation of microbiota-host interactions. However, the evidence is not yet sufficiently standardized to support firm quantitative pharmacological claims for the complete formula. In many studies, IC50, EC50, Ki, minimal active concentration, comparator quality, dose-response relationships, and loss-of-function validation are either absent or inconsistently reported. Table 1 should therefore be interpreted as a qualitative mechanistic map rather than a definitive quantitative pharmacology summary. The relevant content is shown in Figure 2.

**TABLE 1:** Summarizes the major botanical drugs, representative metabolites, and proposed mechanistic directions relevant to BXT.

Botanical drug	Names and species	Representative metabolites	Reported pharmacological direction	Mechanistic targets/pathways and evidence notes
Pinelliae Rhizoma (Zou et al., 2023)	Pinellia ternata (Thunb.) Makino. [Araceae]	Pinelline, alkaloids	Antiemetic, mucosal protection	Inhibition of gastric acid secretion, anti-inflammatory modulation
Coptidis Rhizoma (Sun, 2024)	Coptis chinensis Franch. [Ranunculaceae]	Berberine, coptisine	Anti-inflammatory, anti-proliferative	NF-kB/MAPK-related signaling; mostly metabolite-level evidence rather than complete-formula proof
Scutellariae Radix (Pei et al., 2022)	Scutellaria baicalensis Georgi [Lamiaceae]	Baicalin, baicalein	Antioxidant, apoptotic regulation	p53, Bcl-2/Bax, caspase pathways; evidence largely derived from isolated flavonoids
Zingiberis Rhizoma Recens (Arcusa et al., 2022)	Zingiber officinale Roscoe [Zingiberaceae]	6 Gingerol, shogaol	Gastroprotective, antioxidant	Inhibition of lipid peroxidation, prostaglandin balance
Ginseng Radix et Rhizoma (Zhou et al., 2024)	Panax ginseng C.A.Mey. [Araliaceae]	Ginsenosides Rg1, Rb1	Immunomodulatory, metabolic support	AMPK activation, mitochondrial stabilization
Glycyrrhizae Radix et Rhizoma (Shaikh et al., 2024 )	Glycyrrhiza uralensis Fisch. ex DC. [Fabaceae]	Glycyrrhizin, liquiritin	Anti-ulcer, harmonizing agent	Modulation of cytokine release, glucocorticoid synergy
Jujubae Fructus (Popstoyanova et al., 2024)	Ziziphus jujuba Mill. [Rhamnaceae]	Polysaccharides, flavonoids	Digestive aid, tonic	Enhancement of gut microbiota, mucosal repair

where direct quantitative pharmacological parameters for the complete BXT formula were unavailable, the table retains pathway-oriented summaries and should not be interpreted as proof of formula-level equivalence to isolated-metabolite studies.

Beyond inflammatory control, individual metabolites within BXT - rather than the formula being uniformly proven as a whole - have been linked to pathways involved in angiogenesis, matrix remodeling, and treatment tolerance (Cui et al., 2025). For example, selected ginsenosides or flavonoids may influence VEGF-related signaling or matrix metalloproteinase activity in experimental systems. Such observations support biological plausibility, but they do not by themselves establish that BXT has a clinically verified anti-angiogenic effect in gastric cardia cancer.

From a systemic perspective, BXT’s pharmacological activity reflects the integrative philosophy of Traditional Chinese Medicine (TCM) by treating disease not as a localized pathology but as a disruption within interconnected physiological systems. The formula’s coordinated modulation of digestive, immune, and metabolic networks reflects the comprehensive pattern of disease progression in the cardiac region, characterized by the continuous interaction of inflammation, microbial dysbiosis, and epithelial injury (Gao et al., 2024). In this regard, BXT serves as a prototype for contemporary network-based therapeutics that may connect conventional empirical knowledge and molecular oncology (Feng et al., 2021).

Pharmacokinetic evidence also remains incomplete. Although some BXT-related metabolites undergo phase II metabolism while retaining biological activity, dedicated interaction studies reporting chemotherapy exposure parameters such as AUC or Cmax after BXT co-administration are still insufficient. Claims regarding clinically neutral or beneficial herb-drug interactions should therefore be presented cautiously until standardized pharmacokinetic and pharmacodynamic studies are available.

## 3 Clinical evidence for integrative therapy in gastric cardia cancer and precancerous lesions

Direct clinical evidence specific to gastric cardia cancer is limited, and this limitation should be stated explicitly. Most available clinical reports evaluate BXT in chronic gastritis, reflux-related disease, intestinal metaplasia, dysplasia, or broader upper gastrointestinal oncology settings rather than in rigorously defined gastric cardia cancer cohorts. Accordingly, the current clinical literature supports BXT more strongly as a supportive-care or mucosal-protective intervention than as a proven anti-tumor therapy in gastric cardia cancer (Zhang M. et al., 2021).

## 4 Clinical translation, biomarkers, and remaining gaps

In supportive-care settings, some studies have reported improvement in appetite, nausea, vomiting, epigastric discomfort, or treatment tolerance when BXT-based therapy was combined with standard biomedical treatment (Li L. et al., 2024). However, these studies are heterogeneous in formulation, dose, treatment duration, comparator choice, and endpoint definition. The present evidence therefore suggests possible symptomatic benefit, but not a definitive magnitude of benefit that can be generalized across gastric cardia cancer populations.

For precancerous lesions such as chronic gastritis, intestinal metaplasia, or dysplasia, several reports have described endoscopic or histopathologic improvement after BXT-based intervention, often in combination with acid suppression, *Helicobacter pylori* management, diet modification, or other routine measures (Nishiwaki et al., 2024). These findings are clinically interesting because they align with mechanisms of barrier repair and inflammatory control, but the studies are usually small, variably controlled, and not specific to cardia lesions alone.

Biomarker data should likewise be interpreted cautiously. Some studies describe lower inflammatory or oxidative-stress markers after integrative treatment, yet many do not provide baseline values, post-treatment concentrations, sample sources, assay harmonization, or fully reported statistical comparisons (Feng et al., 2021). As a result, the available biomarker literature is hypothesis-generating rather than confirmatory, and claims about reduction of CRP, IL-6, VEGF, MMP-9, or related markers should be framed as preliminary unless quantitative data are clearly documented.

Immunologic observations such as changes in CD4+/CD8+ balance or natural killer cell activity have also been reported, but the evidence base remains small and methodologically diverse. These data suggest that BXT may influence host immune tone during treatment; they do not yet establish a reproducible immunotherapeutic synergy in gastric cardia cancer.

Safety signals in clinical use appear generally acceptable, with most reported adverse events being mild gastrointestinal complaints. Even so, the current literature does not justify a categorical statement that clinically relevant herb-drug interactions are absent. Dedicated pharmacovigilance datasets and prospective interaction studies are still needed, especially in patients receiving multi-agent chemotherapy, targeted therapy, or immunotherapy.

Overall, the clinical literature is characterized by heterogeneity, modest sample size, incomplete endpoint reporting, and limited long-term oncologic outcomes such as recurrence-free survival, progression-free survival, or overall survival. The most defensible current conclusion is that BXT may have supportive value in selected upper gastrointestinal settings, whereas direct disease-modifying efficacy in gastric cardia cancer remains unproven and requires better-designed multicenter trials.

## 5 Tumor microenvironment, systems biology, and integrative mechanisms of Banxia Xiexin Tang in gastric cardia cancer

Cardia cancer arises in a uniquely hostile and dynamic microenvironment at the gastroesophageal junction, where chronic inflammation, acid and bile reflux, microbial dysbiosis, and mechanical stress converge (Lv et al., 2021; Ko, 2023; Li J. et al., 2024; Ma et al., 2025). Conventional oncology has traditionally focused on malignant epithelial cells as the primary therapeutic target, yet it is increasingly clear that tumor behavior is inseparable from the cellular, molecular, and ecological context in which it develops (Brunner et al., 2022; Créhange et al., 2022; Yang et al., 2022; Ajay et al., 2023; Chen, 2025; Mehrasa and Zamiri, 2025). The tumor microenvironment of the cardia is composed of immune cells, fibroblasts, endothelial cells, extracellular matrix, neural inputs, circulating metabolites, and resident microbiota, all of which interact through dense signaling networks (Yang P. et al., 2023; Kusano et al., 2025; Saadh et al., 2025). These networks regulate angiogenesis, epithelial–mesenchymal transition, invasion, immune evasion, and therapeutic resistance (Liu M. et al., 2021; Yi et al., 2021; Shen et al., 2023). Within this complex systems framework, Banxia Xiexin Tang (BXT) can be conceptualized not as a single drug but as a multi-component regulatory intervention that simultaneously modulates several nodes of the network that underlies cardia carcinogenesis and progression.

### 5.1 Tumor microenvironment and systems-level dysregulation in cardia cancer

Chronic irritation from gastric acid, bile, and dietary carcinogens generates an environment of chronic inflammation, which sets up the tumor microenvironment for cardia cancer by stimulating innate immune responses (TLR-NF-kappaB and inflammasomes), leading to continuous secretion of cytokines (IL-1beta, IL-6 and TNF-alpha). This leads to ongoing oxidative damage, mutagenesis, and degradation of the epithelial barrier, leading to continued loss of barrier integrity (Zhang Z. et al., 2025). With time, the local mucosa will undergo changes from metaplasia and dysplasia to adenocarcinoma, with the tumor microenvironment continuing to support the development and progression of tumor clones (Chen et al., 2024; Marano et al., 2025). In addition to secreting matrix metalloproteinases, growth factors, and chemokines that remodel the extracellular matrix and provide routes for tumor cells to invade the surrounding tissues, the tumor-associated fibroblasts create a pro-tumorigenic microenvironment that supports the survival of malignant clones. Also, the accumulation of tumor-associated macrophages and myeloid-derived suppressive cells in the tumor microenvironment due to chemokine gradients results in the secretion of VEGF, IL-10, and TGF-beta, which facilitates angiogenesis and suppression of adaptive anti-tumor immunity (Janiczek-Polewska et al., 2022; Maxwell et al., 2024).

Additionally, pathways involved in cellular processes (e.g., NF-kappaB, STAT3, Wnt/beta-catenin, PI3K/AKT and MAPK) continue to be active in the tumor microenvironment by soluble signals and cell-to-cell contact between tumor cells and the surrounding cells. As these pathways cooperate, they stimulate proliferation, survival, metabolic reprogramming, and resistance to apoptosis in malignant cells. These pathways also stimulate the expression of molecules involved in the regulation of immune checkpoints (e.g., PD-L1), thereby limiting the activity of cytotoxic T cells (Wang J. et al., 2022; Han et al., 2025; He et al., 2025; Singh and Shukla, 2025). At the same time, alterations to the proteins responsible for maintaining the integrity of the tight junctions (e.g., occludin and claudins) facilitate the back-and-forth movement of microbial products and inflammatory mediators (Wang D. W. et al., 2022; Ouban and Arabi, 2023; Poniewierska-Baran et al., 2025). The microbial populations found in the upper part of the stomach and lower part of the esophagus shift away from a balanced community to a dysbiotic population which is composed primarily of pro-inflammatory bacteria or bacteria capable of generating reactive nitrogen species and which contribute to the increased levels of DNA damage and immune activation (Liu S. et al., 2021; Kim et al., 2022; Chattopadhyay et al., 2023).

Therefore, from a systems perspective, cardia cancer can be viewed as a disease of unstable networks involving the interconnection of epithelial, immune, stromal, vascular, and microbial components in a self-sustaining pathological state. Therefore, single-target therapies, while very effective in disrupting specific molecular pathways, are likely to only temporarily restore homeostasis to the entire system. This explains why some patients experience little clinical benefit or rapidly relapse even after undergoing technically successful local resection or systemic treatment. Additionally, this provides a rationale for developing an integrated therapeutic strategy that targets both molecular pathways and the overall regulatory mechanisms controlling these pathways. Traditional Chinese Medicine, with its focus on diagnosing patterns of disease and treating multiple targets of disease, provides a conceptual basis for understanding how BXT could be used to treat cardia cancer as a whole-system approach to disease.

### 5.2 Integrative mechanisms of Banxia Xiexin Tang in modulating the tumor microenvironment

Banxia Xiexin Tang is made of a number of herbal extracts, each containing substances that affect the various elements of the tumor microenvironment (the inflammatory axis, the epithelial axis, the immune axis, and the metabolic axis) in complementary, additive, and/or cooperative ways. Experimental results show that Banxia Xiexin Tang suppresses the normal inflammatory pathways that include TLR4/NF-kappa-B and NLRP3 inflammasome signaling, and this suppression reduces transcription of IL-6, TNF-alpha, and COX-2 mRNAs. The continuous inflammatory stimulation of the chronic inflammation of the cardia is reduced, and subsequently, the amount of cytokines and mediators produced locally is decreased. Consequently, Banxia Xiexin Tang indirectly diminishes positive feedback loops that maintain the activation of STAT3 and other pro-tumorigenic signaling pathways in epithelial and stromal cells (Zhang X. et al., 2025).

At the same time, Banxia Xiexin Tang appears to help restore the structure and function of the epithelial layer. The upregulation of tight junction proteins such as ZO-1, occludin, and claudins increases the strength of the paracellular seal and limits the diffusion of luminal toxicants, bile acids, and microbial products into the lamina propria. This restoration of the epithelial barrier has two significant implications. First, it reduces the stimulation of chronic immune activation, and consequently, the inflammatory microenvironment is reduced. Second, it limits the diffusion of carcinogenic metabolites into the epithelium, which could slow the progression from metaplasia/dysplasia to overt carcinoma. At the same time, the antioxidant components of Banxia Xiexin Tang improve the activity of superoxide dismutase and glutathione peroxidase, decrease lipid peroxidation, and protect mitochondria in both epithelial and immune cells damaged by reflux (Feng et al., 2021).

Banxia Xiexin Tang’s action is not limited to the epithelial layer. In the stroma, the ginsenoside and flavonoid compounds present in the formula have been demonstrated to inhibit angiogenic signaling by reducing the expression of vascular endothelial growth factor (VEGF) and also to inhibit the proteolytic activity of matrix metalloproteases. These effects may limit the development of new blood vessels and degradation of the extracellular matrix, which are critical processes for tumor expansion and metastasis. In addition, Banxia Xiexin Tang has been linked to the restoration of a more balanced T-cell profile, characterized by increased CD4+/CD8+ ratios and enhanced natural killer (NK) cell function in clinical and preclinical studies. This improved immune tone may, in turn, counteract some of the immunosuppressive signals generated by tumor-associated macrophages and regulatory T-cells.

Another aspect of the tumor microenvironment is the gastric and esophageal microbiota. Recent data suggest that Banxia Xiexin Tang may selectively increase the relative abundance of beneficial bacterial genera (such as *Lactobacillus*) while decreasing the abundance of opportunistic or pro-inflammatory bacteria. By modifying the composition of the gut flora, Banxia Xiexin Tang may also influence the production of microbial metabolites and the presentation of pathogen-associated molecular patterns to mucosal immune cells and epithelial pattern recognition receptors. This type of ecological reorganization should support a shift from a chronic, low-grade activation of innate immunity towards a more regulated state, thereby supporting mucosal healing and limiting neoplastic transformation (Zhang et al., 2022).

These findings collectively demonstrate that Banxia Xiexin Tang acts upon the tumor microenvironment at multiple levels. It includes dampening of inflammatory and pro-tumorigenic signaling pathways; reinforcement of epithelial barriers; modification of oxidative stress and metabolic pathways; remodeling of the microbiota; and calibration of both innate and adaptive immune responses. Therefore, in systems biology terms, Banxia Xiexin Tang represents a network-level intervention that influences the cardia microenvironment to move away from a malignant attractor state and back towards a more homeostatically stable configuration. This would particularly benefit patients if they were treated early in the metaplasia-dysplasia spectrum or as an adjunct to surgery, chemotherapy, or immunotherapy.

### 5.3 Systems biology, translational integration, and a mechanistic framework for BXT

Integrating BXT into modern gastric cardia cancer management within a systems-biology framework is scientifically reasonable only when the formula is treated as a multi-component intervention that acts on interacting networks rather than as a single-target drug. Network pharmacology studies have proposed hub targets such as IL-6, STAT3, CASP3, and PTGS2, but these remain hypothesis-supporting data rather than definitive proof of clinical mechanism.

As a result of the mechanism of action described above, the network-level modulation inherent in BXT aligns well with the concept of “multi-target, low-intensity” intervention, where the goal is to lightly alter complex systems, versus “single-target, high-intensity” interventions, which often induce compensatory resistance mechanisms.

In addition to providing a rationale for incorporating BXT into cardia cancer management, the mechanistic insights provided above provide a number of tangible ways in which BXT could be used to treat cardia cancer. For example, as a preventative agent for patients with chronic gastritis, intestinal metaplasia, or low grade dysplasia, BXT may reduce or even potentially reverse precancerous alterations by increasing epithelial barrier integrity, decreasing oxidative stress, correcting dysbiosis, and/or decreasing inflammatory signaling. Furthermore, as a supportive therapy during the administration of chemotherapy and/or chemoradiotherapy, BXT may protect the gastrointestinal mucosa, decrease the incidence and severity of mucositis and gastritis, and/or decrease systemic inflammation. As a result, BXT may help increase the tolerance of patients to chemotherapy and chemoradiotherapy, improve their nutritional status, and encourage compliance with full dose regimens. With respect to the use of immunotherapy, BXT’s effects on immune checkpoints through IL-6/STAT3 and enhancement of NK-cell and T-cell functions suggest that it may act as a synergistic immunomodulatory adjunct; however, this hypothesis needs to be tested in a rigorously designed trial.

In addition to its translational relevance, BXT may eventually lend itself to biomarker-guided stratification if future studies can link response to inflammatory mediators, microbiome signatures, barrier-integrity markers, or other molecular readouts. At present, however, this remains a forward-looking research agenda rather than an established clinical strategy.

The systems biology perspective on BXT is also relevant for health systems and regulatory agencies. By demonstrating how a traditional formula interacts with modern molecular pathways and impacts clinically relevant outcomes, the incorporation of BXT into integrative treatment guidelines can be more easily justified. Furthermore, designing clinical trials with appropriate mechanistic and clinical endpoints can be facilitated by understanding how a traditional formula acts on modern molecular pathways. Additionally, converting BXT from a decoction to a standardized granule, capsule, or injectable formulation will require a thorough characterization of its pharmacologically active spectrum and quality control across batches. Systems biology can assist in this process by identifying those components and combinations of components that are most critical for modulating the microenvironment, thus aiding in the rational standardization of BXT, rather than simply reproducing the traditional ratio empirically.

To illustrate these complex relationships in a straightforward fashion, it is useful to compare the typical state of the cardia tumor microenvironment in the absence of BXT with its predicted state when BXT-based integrative therapy is administered. The following table illustrates five distinct aspects of the cardia tumor microenvironment—epithelial barrier function, inflammatory signaling, microbiota composition, vascular and stromal remodeling, and immune tone—and describes how each of these aspects is commonly modified in cardia cancer, as well as how BXT is expected to modify each of these aspects based on available preclinical and clinical data. This systematic comparison demonstrates the systems-level nature of the actions of BXT and serves to clarify how a single traditional prescription may act to affect multiple therapeutic targets in a coherent and biologically plausible manner. The relevant summary of Chapter 5 can be found in Table 2.

**TABLE 2:** Comparative overview of tumor microenvironment features in gastric cardia cancer with and without BXT-based integrative therapy, compiled from the studies discussed in Sections 5.1–5.3.

Microenvironmental domain	Typical features in cardia cancer without BXT	Proposed modifications under BXT-Based integrative therapy
Epithelial barrier integrity	Disruption of tight junctions; increased permeability, and facilitated translocation of bile acids, acids, and microbial products	Upregulation of tight junction proteins; improved mucosal sealing, and reduced paracellular leakage of luminal toxins and carcinogens
Inflammatory signaling and cytokine milieu	Persistent activation of TLR/NF-κB and inflammasome pathways; elevated IL-1β, IL-6, TNF-α, and COX-2 expression	Attenuation of TLR4/NF-κB signaling; decreased pro-inflammatory cytokines and COX-2; partial normalization of mucosal inflammatory tone
Oxidative stress and metabolic status	Excess reactive oxygen species; lipid peroxidation; mitochondrial dysfunction, and metabolic shift favoring tumor survival	Enhanced antioxidant enzyme activity, reduced lipid peroxidation, stabilization of mitochondrial function, and partial correction of metabolic stress
Microbiota composition	Dysbiosis with expansion of pro-inflammatory or nitrosating bacteria; increased production of harmful metabolites	Relative increase in beneficial commensals (e.g., *Lactobacillus*); reduction of pathogenic species; altered metabolite profile supporting mucosal repair
Stromal and vascular remodeling	Activation of cancer-associated fibroblasts; elevated MMP activity, and VEGF-driven neoangiogenesis facilitating invasion and metastasis	Suppression of VEGF and MMP expression in experimental models; potential reduction in aberrant angiogenesis and matrix degradation
Immune tone and anti-tumor surveillance	Accumulation of immunosuppressive cells, high expression of immune checkpoints, and impaired cytotoxic T-cell and NK-cell function	Improved CD4^+^/CD8^+^ balance, enhanced NK activity, indirect modulation of checkpoint-related pathways, and partial restoration of anti-tumor immunity
Clinical treatment tolerance and symptom burden	High rates of mucositis, gastritis, nausea, and anorexia during chemotherapy; reduced adherence to full-dose regimens	Reduced gastrointestinal toxicity; improved appetite and nutritional status; better tolerance of chemotherapy and potential completion of planned regimens

## 6 Safety, herb–drug interactions, and quality control considerations

Safety is a central requirement for any integrative oncology strategy. For BXT, the main safety questions concern formulation standardization, raw-material authentication, contamination control, and potential herb-drug interactions during concurrent oncologic therapy. The revised interpretation therefore prioritizes cautious compatibility rather than assuming universal safety across all treatment settings.

Available clinical reports generally describe BXT as reasonably well tolerated, with most adverse reactions limited to mild gastrointestinal symptoms. However, many safety datasets are small, short-term, and not designed to detect rare adverse events, delayed toxicity, or interactions under complex oncology regimens. The absence of strong safety signals should not be interpreted as proof of absence of risk.

Potential herb-drug interactions deserve explicit attention because metabolites such as berberine, baicalin, and ginsenosides may affect transporters or metabolizing enzymes relevant to anticancer drugs. At present, dedicated clinical interaction studies measuring exposure changes of chemotherapy agents during BXT co-administration are insufficient. Accordingly, concomitant use should be supervised clinically, especially when narrow-therapeutic-index drugs are involved.

In contrast to many other single herbal extracts, the internal safety of BXT is far superior due to the synergism of the individual ingredients, which mutually inhibit one another’s toxicity. Each ingredient (for instance, ginger) counteracts any irritation it may cause to the mucous membranes (as caused by Pinellia ternata), while Glycyrrhiza uralensis acts in a complementary manner to decrease gastrointestinal side effects and regulate the body-wide absorption of each ingredient. These built-in buffer systems are founded upon the principle of “mutual detoxification” found within Traditional Chinese Medicine and represent a major aspect of creating TCM formulas (Zhang et al., 2024). While they provide an additional layer of safety as pharmacologically active agents, if the balance of herb ratios is disrupted by the inappropriate use of self-medication or through unregulated alterations of those ratios, the potential exists for unpredictable results; thus, the need for practitioner oversight (Shi et al., 2024).

There is a need for clinical integration that recognizes the regulatory frameworks related to pharmacovigilance for herbal medicines in order to have a long-term view of adverse events (i.e., monitor adverse events; maintain records of herb-drug interactions, and establish registries for integrative therapies). Agreement by Eastern and Western health systems regarding standards for safe practices of integrative oncology will assist the growth of integrative oncology as a global practice. Standardized reporting systems such as VigiBase of the World Health Organization for pharmacovigilance of herbs can facilitate clarity and enable international sharing of data (Sun et al., 2021).

Additionally, it’s really important to differentiate between BXT decoctions manufactured with certified ingredients and commercial versions or generic/over-the-counter (OTC) versions of BXT that can contain different formulation, impurities, etc (Ngum et al., 2023). The literature indicates that some formulations may be modified or substituted with inappropriate substitutes or adulterants that will diminish the effectiveness of BXT while increase the risk of toxic effects; this issue is particularly relevant to international patients who purchase herbal products outside of regulated pharmaceutical systems. Therefore, in order to ensure safety as well as consistent clinical effect of BXT, it is essential that it is only provided by certified health professionals and produced by GMP-compliant manufacturers (Xu et al., 2021).

A safe and evidence-based role for BXT in patients with gastric cardia cancer is therefore possible only under conditions of clinician oversight, standardized product quality, and transparent monitoring for adverse events and interactions. Its use should be framed as adjunctive, individualized, and evidence-limited rather than universally interchangeable with routine oncologic supportive care.

## 7 Implementation strategies and future perspectives

Implementing BXT in gastric cardia cancer care requires a translational framework that aligns traditional practice with oncology standards for diagnosis, product quality, safety monitoring, endpoint reporting, and multidisciplinary decision-making. The goal is not rhetorical integration, but clinically accountable integration.

Multidisciplinary collaboration is required for the integration of BXT in clinical practice. The best treatment outcomes result from collaborative development of treatment plans by oncologists, pharmacologists, and TCM practitioners that incorporate the individual patient’s unique needs and requirements as well as the optimal timing and interaction between drugs (Zu et al., 2024). For example, BXT could also be employed as part of an overall strategy for the treatment of gastrointestinal toxicity that occurs during the course of chemotherapy, or could be employed post-surgically to improve the rate of mucosal repair and metabolic stabilization. BXT does not simply treat the symptoms; rather, through the control of inflammation, immune responses, and gastric microbiota, it is able to provide balance at the cellular level when combined with Western pharmaceuticals. The concept of “treat the root and the branches” is fundamental to the principles of TCM (Traditional Chinese Medicine) and is currently receiving support from molecular data regarding dual therapeutic strategies (Wei et al., 2021).

In addition to implementation strategies, integrative clinical pathways need to be developed that provide clarity on how herbal medicine (such as BXT) will be ordered by hospital-based oncology departments. Pharmacognostic verification of the correct dose needs to occur at the same time that the correct dose of BXT is verified. Multidisciplinary tumor boards across many integrative oncology units in China have set a precedent for the model being described above; the discussion of herbal formulations is an integral part of these multidisciplinary tumor board discussions when considering surgery, radiotherapy, and immunotherapy. If the model of integrative oncology units in China is adopted around the world, then complementary therapy may shift from being an optional add-on to being a necessary component of comprehensive cancer treatment (Kim et al., 2021).

A big part of implementing a global program will involve modernization and standardization of how BXT is prepared. Because there are many different methods of obtaining, processing, and extracting the herbs used in BXT, it is difficult to predict the level of pharmacologic activity or therapeutic outcome. Future development must include modern formulations such as granules, capsules, and standardized extracts that maintain the synergistic activity of the traditional decoction while providing a predictable dose. Additionally, education and training will be critical for long term integration of BXT into clinical practice. Most Western-trained oncologists do not have knowledge of Traditional Chinese Medicine (TCM) principles and most TCM practitioners lack knowledge of the pharmacokinetic and molecular mechanisms underlying Western chemotherapy and radiation. An equally significant challenge will be developing a better understanding of BXT’s bioactive spectrum through advanced analytical technologies, including ultra-performance liquid chromatography (UPLC), metabolomics, and systems pharmacology, so that BXT is reliable for clinical use (Luo et al., 2023). The establishment of education programs that combine professionals from various disciplines (research fellowships and hospital exchanges) will help to provide the next-generation of an integrative clinician educated in both paradigms. The synergy of these professionals will be in ensuring that decisions regarding treatments involving combination herbal drugs are science-based, ethical and patient centered (Zhang Y. et al., 2021).

Further studies will need to move from observational trials to large-scale randomized controlled trials using uniform diagnostic criteria and quantitative biomarkers. The symptomatic and supportive benefits of BXT have been well-documented; however, long-term implications for survival, recurrence, and manipulation of the tumor microenvironment are poorly characterized. Use of omics technologies (genomics, transcriptomics, proteomics, and metabolomics) in clinical studies can provide insight into the molecular mechanisms underlying BXT’s multi-targeted action and identify potential predictive biomarkers for response to BXT treatment. Such knowledge will transition BXT from an adjuvant treatment option to a scientifically supported component of personalized oncology.

From a regulatory standpoint, inclusion of BXT as part of standard treatments will require a unifying framework of regulation among all countries’ health authorities. It is essential to establish pharmacopeial standards for contamination testing, authentication of herbal materials, and quantification of bioactive compounds. International cooperation, such as collaborative agreements between China’s regulatory agencies and those of the United States (FDA), Europe (EMA) and World Health Organization (WHO), could enable cross-country recognition of pharmacologic data regarding herbs, thus facilitating global acceptance of integrative therapies. Ultimately, the inclusion of BXT in official guidelines for cancer treatment based on high-quality evidence would represent a significant advancement towards the routine integration of integrative cancer care into medical practice.

The integration of digital technology and artificial intelligence may further facilitate the application of integrative medicine in the clinical setting. Artificial intelligence-driven network pharmacology may provide real-time mapping of interactions between herbal compounds and cellular targets. Additionally, machine learning algorithms developed and trained with both clinical and molecular data may allow for the stratification of patients who may derive the greatest benefit from BXT-based therapy. This type of data-driven personalized approach aligns with the future vision of precision integrative medicine, which aims to apply modern computational methods to ancient formulae.

Public health-wise, the availability of low-cost, easily accessible adjuncts to conventional cancer treatment through integrative approaches may help address treatment inequities. Standardized and safe herbal treatments can decrease healthcare costs and reduce the secondary effects of conventional treatment that frequently result in costly hospitalizations and premature discontinuation of treatment. Thus, incorporating TCM into oncology systems is not merely a cultural convergence; it is also a practical means of reaching and improving the wellbeing of additional patients.

## 8 Conclusion

The integration of Traditional Chinese Medicine and standard biomedical treatment offers a potentially valuable framework for the management of gastric cardia cancer and related precancerous lesions, but the evidence must be interpreted with precision. Banxia Xiexin Tang is biologically interesting because it intersects with pathways relevant to inflammation, oxidative stress, mucosal injury, microbiota imbalance, and treatment tolerance. At the same time, the current literature does not yet justify strong claims of direct anti-cancer efficacy in gastric cardia cancer.

Banxia Xiexin Tang (BXT) is a paradigm of synergistic therapy for which scientific evidence supports the traditional empirical basis of the formulation, providing a mechanism of action that validates the pharmacological effectiveness of the formulation in addition to empirical precedent.

Taken together, the reviewed data support BXT primarily as a candidate adjunctive intervention with possible supportive, mucosal-protective, and host-regulatory benefits. The strongest themes in the literature are improvement in upper gastrointestinal symptoms, protection of epithelial integrity, and modulation of inflammatory or oxidative pathways in preclinical or non-cardia settings. By contrast, evidence for survival benefit, recurrence reduction, or direct tumor control in gastric cardia cancer remains insufficient.

Future work should therefore emphasize rigorous study design, disease-specific cohorts, standardized BXT preparations, transparent dose and comparator reporting, quantitative biomarker assessment, herb-drug interaction studies, and multicenter trials with clinically meaningful oncologic endpoints. Only through such work can BXT be positioned credibly within evidence-based integrative oncology.

The therapeutic philosophy of Banxia Xiexin Tang represents the evolving model of 21st-century medicine, defined by the integration of exacting science and balanced wholeness. The integration of molecular oncology with the systemic wisdom of traditional pharmacology defines cancer therapy as an adaptable, patient-centered, and integrated approach to comprehensive cancer care. Ultimately, the path forward is not to supplant one model of care with another but to integrate the best aspects of each to develop a more inclusive and effective model of healthcare.
